# Spectrum and Frequency of Germline *FANCM* Protein-Truncating Variants in 44,803 European Female Breast Cancer Cases

**DOI:** 10.3390/cancers15133313

**Published:** 2023-06-23

**Authors:** Gisella Figlioli, Amandine Billaud, Qin Wang, Manjeet K. Bolla, Joe Dennis, Michael Lush, Anders Kvist, Muriel A. Adank, Thomas U. Ahearn, Natalia N. Antonenkova, Päivi Auvinen, Sabine Behrens, Marina Bermisheva, Natalia V. Bogdanova, Stig E. Bojesen, Bernardo Bonanni, Thomas Brüning, Nicola J. Camp, Archie Campbell, Jose E. Castelao, Melissa H. Cessna, NBCS Collaborators, Kamila Czene, Peter Devilee, Thilo Dörk, Mikael Eriksson, Peter A. Fasching, Henrik Flyger, Marike Gabrielson, Manuela Gago-Dominguez, Montserrat García-Closas, Gord Glendon, Encarna B. Gómez Garcia, Anna González-Neira, Felix Grassmann, Pascal Guénel, Eric Hahnen, Ute Hamann, Peter Hillemanns, Maartje J. Hooning, Reiner Hoppe, Anthony Howell, Keith Humphreys, kConFab Investigators, Anna Jakubowska, Elza K. Khusnutdinova, Vessela N. Kristensen, Annika Lindblom, Maria A. Loizidou, Jan Lubiński, Arto Mannermaa, Tabea Maurer, Dimitrios Mavroudis, William G. Newman, Nadia Obi, Mihalis I. Panayiotidis, Paolo Radice, Muhammad U. Rashid, Valerie Rhenius, Matthias Ruebner, Emmanouil Saloustros, Elinor J. Sawyer, Marjanka K. Schmidt, Rita K. Schmutzler, Mitul Shah, Melissa C. Southey, Ian Tomlinson, Thérèse Truong, Elke M. van Veen, Camilla Wendt, Xiaohong R. Yang, Kyriaki Michailidou, Alison M. Dunning, Paul D. P. Pharoah, Douglas F. Easton, Irene L. Andrulis, D. Gareth Evans, Antoinette Hollestelle, Jenny Chang-Claude, Roger L. Milne, Paolo Peterlongo

**Affiliations:** 1 Genome Diagnostics Program, IFOM ETS—The AIRC Institute of Molecular Oncology, 20139 Milan, Italy; gisella.figlioli@ifom.eu (G.F.);; 2 Centre for Cancer Genetic Epidemiology, Department of Public Health and Primary Care, University of Cambridge, Cambridge CB1 8RN, UK; 3 Division of Oncology, Department of Clinical Sciences Lund, Lund University, 22185 Lund, Sweden; 4 The Netherlands Cancer Institute—Antoni van Leeuwenhoek Hospital, Family Cancer Clinic, 1066 CX Amsterdam, The Netherlands; 5 Division of Cancer Epidemiology and Genetics, National Cancer Institute, National Institutes of Health (NIH), Department of Health and Human Services, Bethesda, MD 20892, USA; 6 N.N. Alexandrov Research Institute of Oncology and Medical Radiology, 223040 Minsk, Belarus; 7 Translational Cancer Research Area, University of Eastern Finland, 70210 Kuopio, Finland; 8 Institute of Clinical Medicine, Oncology, University of Eastern Finland, 70210 Kuopio, Finland; 9 Department of Oncology, Cancer Center, Kuopio University Hospital, 70210 Kuopio, Finland; 10 Division of Cancer Epidemiology, German Cancer Research Center (DKFZ), 69120 Heidelberg, Germany; 11 Institute of Biochemistry and Genetics of the Ufa Federal Research Centre of the Russian Academy of Sciences, 450054 Ufa, Russia; 12 Department of Radiation Oncology, Hannover Medical School, 30625 Hannover, Germany; 13 Gynaecology Research Unit, Hannover Medical School, 30625 Hannover, Germany; 14 Copenhagen General Population Study, Copenhagen University Hospital, Herlev and Gentofte Hospital, 2730 Herlev, Denmark; 15 Department of Clinical Biochemistry, Copenhagen University Hospital, Herlev and Gentofte Hospital, 2730 Herlev, Denmark; 16 Faculty of Health and Medical Sciences, University of Copenhagen, 2200 Copenhagen, Denmark; 17 Division of Cancer Prevention and Genetics, IEO, European Institute of Oncology IRCCS, 20141 Milan, Italy; 18 Institute for Prevention and Occupational Medicine of the German Social Accident Insurance, Institute of the Ruhr University Bochum (IPA), 44789 Bochum, Germany; 19 Department of Internal Medicine and Huntsman Cancer Institute, University of Utah, Salt Lake City, UT 84112, USA; 20 Centre for Genomic and Experimental Medicine, Institute of Genetics & Cancer, University of Edinburgh, Edinburgh EH4 2XU, UK; 21 Usher Institute of Population Health Sciences and Informatics, University of Edinburgh, Edinburgh EH16 4UX, UK; 22 Oncology and Genetics Unit, Instituto de Investigación Sanitaria Galicia Sur (IISGS), Xerencia de Xestion Integrada de Vigo-SERGAS, 36312 Vigo, Spain; 23 Intermountain Health, Salt Lake City, UT 84111, USA; 24 Department of Cancer Genetics, Institute for Cancer Research, Oslo University Hospital-Radiumhospitalet, 0379 Oslo, Norway; 25 Institute of Clinical Medicine, Faculty of Medicine, University of Oslo, 0450 Oslo, Norway; 26 Department of Research, Vestre Viken Hospital, 3019 Drammen, Norway; 27 Biostatistics Unit, The Cyprus Institute of Neurology & Genetics, 2371 Nicosia, Cyprus; 28 Section for Breast- and Endocrine Surgery, Department of Cancer, Division of Surgery, Cancer and Transplantation Medicine, Oslo University Hospital-Ullevål, 0450 Oslo, Norway; 29 Department of Radiology and Nuclear Medicine, Oslo University Hospital, 0379 Oslo, Norway; 30 Department of Pathology, Akershus University Hospital, 1478 Lørenskog, Norway; 31 Department of Tumor Biology, Institute for Cancer Research, Oslo University Hospital, 0379 Oslo, Norway; 32 Department of Oncology, Division of Surgery, Cancer and Transplantation Medicine, Oslo University Hospital-Radiumhospitalet, 0379 Oslo, Norway; 33 National Advisory Unit on Late Effects after Cancer Treatment, Oslo University Hospital, 0379 Oslo, Norway; 34 Department of Oncology, Akershus University Hospital, 1478 Lørenskog, Norway; 35 Oslo Breast Cancer Research Consortium, Oslo University Hospital, 0379 Oslo, Norway; 36 Department of Medical Epidemiology and Biostatistics, Karolinska Institutet, 171 76 Stockholm, Sweden; 37 Department of Pathology, Leiden University Medical Center, 2333 ZA Leiden, The Netherlands; 38 Department of Human Genetics, Leiden University Medical Center, 2333 ZA Leiden, The Netherlands; 39 Department of Gynecology and Obstetrics, University Hospital Erlangen, Comprehensive Cancer Center Erlangen-EMN, Friedrich-Alexander University Erlangen-Nuremberg, 91054 Erlangen, Germany; 40 Department of Breast Surgery, Copenhagen University Hospital, Herlev and Gentofte Hospital, 2730 Herlev, Denmark; 41 Instituto de Investigación Sanitaria de Santiago de Compostela (FIDIS) Foundation, IDIS Cancer Genetics and Epidemiology Group, Genomic Medicine Group, Complejo Hospitalario Universitario de Santiago, SERGAS, 15706 Santiago de Compostela, Spain; 42 Lunenfeld-Tanenbaum Research Institute of Mount Sinai Hospital, Fred A. Litwin Center for Cancer Genetics, Toronto, ON M5G 1X5, Canada; 43 Laboratory Medicine Program, University Health Network, Toronto, ON M5G 2C4, Canada; 44 Department of Clinical Genetics, Maastricht University Medical Center, 6229 HX Maastricht, The Netherlands; 45 Human Genotyping Unit-CeGen, Spanish National Cancer Research Centre (CNIO), 28029 Madrid, Spain; 46 Department of Medicine, Institute for Clinical Research and Systems Medicine, Health and Medical University, 14467 Potsdam, Germany; 47 CESP U1018, Inserm “Exposome, Heredity, Cancer and Health” Team, UVSQ, University Paris-Saclay, Gustave Roussy, 94805 Villejuif, France; 48 Center for Familial Breast and Ovarian Cancer, Faculty of Medicine and University Hospital Cologne, University of Cologne, 50937 Cologne, Germany; 49 Center for Integrated Oncology (CIO), Faculty of Medicine and University Hospital Cologne, University of Cologne, 50937 Cologne, Germany; 50 German Cancer Research Center (DKFZ), Molecular Genetics of Breast Cancer, 69120 Heidelberg, Germany; 51 Department of Medical Oncology, Erasmus MC Cancer Institute, 3015 GD Rotterdam, The Netherlands; 52 Dr. Margarete Fischer-Bosch-Institute of Clinical Pharmacology, 70376 Stuttgart, Germany; 53 University of Tübingen, 72074 Tübingen, Germany; 54 Division of Cancer Sciences, University of Manchester, Manchester M13 9PL, UK; 55 Research Department, Peter MacCallum Cancer Center, Melbourne, VIC 3000, Australia; 56 Sir Peter MacCallum Department of Oncology, The University of Melbourne, Parkville, VIC 3000, Australia; 57 Department of Genetics and Pathology, International Hereditary Cancer Center, Pomeranian Medical University in Szczecin, 71-252 Szczecin, Poland; 58 Independent Laboratory of Molecular Biology and Genetic Diagnostics, Pomeranian Medical University in Szczecin, 71-252 Szczecin, Poland; 59 Department of Genetics and Fundamental Medicine, Ufa University of Science and Technology, 450076 Ufa, Russia; 60 Department of Medical Genetics, Oslo University Hospital and University of Oslo, 0379 Oslo, Norway; 61 Department of Molecular Medicine and Surgery, Karolinska Institutet, 171 76 Stockholm, Sweden; 62 Department of Clinical Genetics, Karolinska University Hospital, 171 76 Stockholm, Sweden; 63 Department of Cancer Genetics, Therapeutics and Ultrastructural Pathology, The Cyprus Institute of Neurology & Genetics, 2371 Nicosia, Cyprus; 64 Institute of Clinical Medicine, Pathology and Forensic Medicine, University of Eastern Finland, 70210 Kuopio, Finland; 65 Kuopio University Hospital, Biobank of Eastern Finland, 70210 Kuopio, Finland; 66 Cancer Epidemiology Group, University Cancer Center Hamburg (UCCH), University Medical Center Hamburg-Eppendorf, 20246 Hamburg, Germany; 67 Department of Medical Oncology, University Hospital of Heraklion, 711 10 Heraklion, Greece; 68 Division of Evolution, Infection and Genomics, School of Biological Sciences, Faculty of Biology, Medicine and Health, University of Manchester, Manchester M13 9PL, UK; 69 Manchester Centre for Genomic Medicine, St. Mary’s Hospital, Manchester University NHS Foundation Trust, Manchester Academic Health Science Centre, Manchester M13 9WL, UK; 70 University Medical Center Hamburg-Eppendorf, Institute for Medical Biometry and Epidemiology, 20246 Hamburg, Germany; 71 Unit of ‘Predictive Medicine: Molecular Bases of Genetic Risk’, Department of Experimental Oncology, Fondazione IRCCS Istituto Nazionale dei Tumori (INT), 20133 Milan, Italy; 72 Department of Basic Sciences, Shaukat Khanum Memorial Cancer Hospital and Research Centre (SKMCH & RC), Lahore 54000, Pakistan; 73 Centre for Cancer Genetic Epidemiology, Department of Oncology, University of Cambridge, Cambridge CB1 8RN, UK; 74 Department of Oncology, University Hospital of Larissa, 411 10 Larissa, Greece; 75 King’s College London, School of Cancer & Pharmaceutical Sciences, Comprehensive Cancer Centre, Guy’s Campus, London SE1 9RT, UK; 76 Division of Molecular Pathology, The Netherlands Cancer Institute, 1066 CX Amsterdam, The Netherlands; 77 Division of Psychosocial Research and Epidemiology, The Netherlands Cancer Institute-Antoni van Leeuwenhoek Hospital, 1066 CX Amsterdam, The Netherlands; 78 Department of Clinical Genetics, Leiden University Medical Center, 2333 ZA Leiden, The Netherlands; 79 Center for Molecular Medicine Cologne (CMMC), Faculty of Medicine and University Hospital Cologne, University of Cologne, 50931 Cologne, Germany; 80 Precision Medicine, School of Clinical Sciences at Monash Health, Monash University, Clayton, VIC 3168, Australia; 81 Department of Clinical Pathology, The University of Melbourne, Melbourne, VIC 3000, Australia; 82 Cancer Epidemiology Division, Cancer Council Victoria, Melbourne, VIC 3004, Australia; 83 Cancer Research Centre, The University of Edinburgh, Edinburgh EH4 2XU, UK; 84 Department of Clinical Science and Education, Karolinska Institutet, Södersjukhuset, 118 83 Stockholm, Sweden; 85 Department of Computational Biomedicine, Cedars-Sinai Medical Center, West Hollywood, CA 90069, USA; 86 Department of Molecular Genetics, University of Toronto, Toronto, ON M5S 1A8, Canada; 87 Centre for Epidemiology and Biostatistics, Melbourne School of Population and Global Health, The University of Melbourne, Melbourne, VIC 3010, Australia

**Keywords:** breast cancer predisposition, breast cancer risk factors, *FANCM* PTVs spectrum, protein truncating variants, PTVs

## Abstract

**Simple Summary:**

Mutations in the *FANCM* gene may cause a particular type of breast cancer known as ER-negative. In this study, we describe the geographic distribution of 66 different *FANCM* mutations identified in 44,803 female breast cancer cases from Europe, USA, Canada and Australia. We found that the *FANCM*:p.Gln1701* mutation is most common in Northern Europe and has lower frequencies in Southern European countries. In contrast, the *FANCM*:p.Gly1906Alafs*12 mutation is most common in Southern Europe and rarer in Central and Northern Europe. We found that the *FANCM*:p.Arg658* mutation is most prevalent in Central Europe and that the *FANCM*:p.Gln498Thrfs*7 mutation originates from Lithuania. Finally, we showed that many and varied *FANCM* mutations are present in Southwestern and Central Europeans while a much more limited range of mutations is present in Northeastern Europeans. The knowledge of this geographic distribution of *FANCM* mutations is important to establish more efficient genetic testing strategies in specific populations.

**Abstract:**

*FANCM* germline protein truncating variants (PTVs) are moderate-risk factors for ER-negative breast cancer. We previously described the spectrum of *FANCM* PTVs in 114 European breast cancer cases. In the present, larger cohort, we report the spectrum and frequency of four common and 62 rare *FANCM* PTVs found in 274 carriers detected among 44,803 breast cancer cases. We confirmed that p.Gln1701* was the most common PTV in Northern Europe with lower frequencies in Southern Europe. In contrast, p.Gly1906Alafs*12 was the most common PTV in Southern Europe with decreasing frequencies in Central and Northern Europe. We verified that p.Arg658* was prevalent in Central Europe and had highest frequencies in Eastern Europe. We also confirmed that the fourth most common PTV, p.Gln498Thrfs*7, might be a founder variant from Lithuania. Based on the frequency distribution of the carriers of rare PTVs, we showed that the *FANCM* PTVs spectra in Southwestern and Central Europe were much more heterogeneous than those from Northeastern Europe. These findings will inform the development of more efficient *FANCM* genetic testing strategies for breast cancer cases from specific European populations.

## 1. Introduction

Breast cancer is a common disease in which up to 25% of the cases are expected to be caused by genetic risk factors [1]. Germline pathogenic variants in the *BRCA1* and *BRCA2* genes are associated with high risks of developing breast cancer. Specifically, the cumulative risks for the disease by age 80 were estimated to be 72% and 69% in women with a *BRCA1* or *BRCA2* pathogenic variant, respectively [2]. Since the identification of *BRCA1* and *BRCA2* thirty years ago, many other genes have been proposed to be associated with moderate to high risk for breast cancer; however, limited and sometimes contradictory findings from studies have impeded a conclusive annotation. In 2020, modified segregation analyses performed in 524 breast cancer families with pathogenic variants in *PALB2* confirmed that this gene confers a risk for breast cancer that is comparable to that of *BRCA2* [3]. One year later, two very large association studies were conducted, and several known and putative predisposition genes were sequenced in a total of more than 178,000 female breast cancer cases and controls [4,5]. The unprecedented statistical power of such large datasets enabled confirmation that protein-truncating variants (PTVs) in *BRCA1*, *BRCA2*, *PALB2* and the Li-Fraumeni syndrome gene *TP53* confer high risk for breast cancer. In addition, these data clarified that PTVs in *BARD1*, *RAD51C* and *RAD51D* are associated with moderate risk of estrogen receptor ER-negative breast cancer and that PTVs in *ATM* and *CHEK2* are associated with moderate risk of ER-positive breast cancer [4,5].

The above-mentioned breast cancer predisposition genes have been tested worldwide and many founder variants, and variants prevalent in specific ethnic or geographic groups, have been described. This knowledge could be used to inform first pass genetic screening and more efficient strategies for genetic testing in specific populations. The prevalence and spectrum of *BRCA1* and *BRCA2* pathogenic variants have been reported in many different populations. Probably the two largest studies conducted so far in these genes are based one on pathogenic variants found in more than 29,000 families from 49 countries, and the other in families from the Middle East, North Africa and Southern Europe [6,7]. Comprehensive analyses of the mutational spectra of *PALB2*, *BARD1*, *RAD51C* and *RAD51D* were described in three systematic reviews including 151 [8], 123 [9] and 101 [10] studies. Finally, a study of the mutational spectrum of *CHEK2* pathogenic variants was recently conducted, but was limited to the Baltic states [11].

While there is a consensus that the genes to be screened to predict the individual risk for breast cancer in diagnostic setting should be *BRCA1*, *BRCA2*, *PALB2*, *TP53*, *BARD1*, *RAD51C*, *RAD51D*, *ATM* and *CHEK2*, other predisposing genes, such as *FANCM*, are yet to be validated [12]. Burden analyses derived from *FANCM* sequencing, but also genotyping of the single most common variants, have shown that *FANCM* PTVs are generally associated with ER-negative or triple-negative breast cancer (TNBC, reviewed in [13]). In particular, the strongest association for these disease subtypes in Europeans is with the common p.Arg658* (c.1972C>T) variant, which truncates the 2048 amino acid FANCM protein at the N-terminus [14]. The risks associated with the other common *FANCM* PTVs p.Gln1701* (c.5101C>T) and p.Gly1906Alafs*12 (c.5791C>T, also known as p.Arg1931* [15]), which truncate the FANCM protein at the C-terminus, appear, in Europeans, to be of lower magnitude or have not been conclusively assessed. However, p.Gln1701* and p.Gly1906Alafs*12 PTVs have been associated with risk for ER-negative and TNBC subtypes in Finnish women [16,17], which we speculate might be due to population-specific variants acting as risk modifiers [13].

We previously described the spectrum of 27 different *FANCM* germline PTVs found in 114 female breast cancer cases ascertained from 13 European countries [18]. In the present study, we analyzed *FANCM* sequencing data from 44,803 female breast cancer cases from 16 European countries and from USA, Canada and Australia, reported the frequency of PTV carriers, and described the spectrum of the 66 different *FANCM* germline PTVs that were found in the 274 carriers.

## 2. Materials and Methods

The 44,803 breast cancer cases included in the present analysis were originally ascertained by 39 studies from 16 European countries and from USA, Canada and Australia that participated in the BRIDGES study (https://bridges-research.eu/, Supplementary Table S1). All these breast cancer cases were women of European ancestry and were older than 18 years at breast cancer diagnosis. Women carrying a pathogenic variant in the *BRCA1* and/or *BRCA2* genes were excluded from the study. All the 44,803 breast cancer cases underwent complete sequencing of the *FANCM* coding region and intron/exon boundaries in the context of the BRIDGES study [4]. Details of the library preparation, sequencing, variant calling and quality control methods have been described elsewhere [4]. Germline *FANCM* PTVs were defined as frameshift or nonsense variants. As a proxy for the carrier’s or PTV’s geographical origin, we used the country where the study ascertaining the carrier was conducted. PTV carrier frequencies were compared using Pearson’s chi-squared test, all tests were two-sided. *p*-values < 0.05 were considered statistically significant.

## 3. Results and Discussion

### 3.1. Frequency of Germline FANCM PTVs

**Figure 1:**
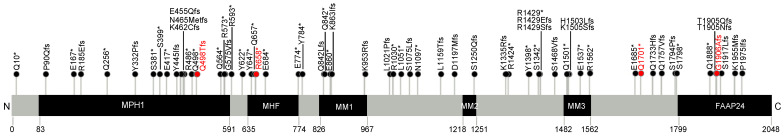
Schematic representation of the 66 *FANCM* protein truncating variants (PTVs) with respect to functional and binding domains (in black). The exact positions of these domains (MPH1, ATP-dependent DNA helicase; MHF, domain of interaction with the Histone Fold 1 and 2 (MHF1/2); MM1, motif of interaction with FANCF within the Fanconi Anemia core complex; MM2, motif of interaction with RecQ-Mediated genome Instability protein 1 (RMI1); MM3, highly conserved motif of still unknown function; FAAP24, domain of interaction with the Fanconi Anemia core complex-Associated Protein 24) were derived from to UniProt database and the published literature [21,22,23]. The 62 rare PTVs and the 4 common PTVs are shown in black and red, respectively. The protein N-terminus (N) and C-terminus (C) are also indicated.

Sixty-six different *FANCM* PTVs were found in 274 PTV carriers that were identified by gene sequencing of 44,803 female breast cancer cases (Figure 1, and Supplementary Table S2, and Table 1). A large percentage (65.3%) of the carriers carried either p.Gln1701* or p.Gly1906Alafs*12. Importantly, for these two PTVs the evidence of association with breast cancer risk was previously inconclusive [13]. Thus, we studied the frequencies of the carriers of all PTVs and of the carriers of all PTVs excluding p.Gln1701* and p.Gly1906Alafs*12. The frequencies of carriers of all PTVs in the 19 tested countries were heterogeneous, varying between 2.50% in Finland and 0.20% in Canada. However, the exclusion of p.Gln1701* or p.Gly1906Alafs*12 carriers resulted in PTV carrier frequencies which were more homogeneous, ranging between 0.11% in France and 0.63% in Belarus (Table 1). We also compared the frequencies of the two groups of PTV carriers with respect to their breast cancer family history and the ER status of their tumors. In these analyses, we observed a significantly higher PTV carrier frequency in familial versus sporadic cases (*p*-value = 0.032), and in ER-negative versus ER-positive cases (*p*-value = 0.048). When we excluded carriers of p.Gln1701* and p.Gly1906Alafs*12, these differences became greater in both familial versus sporadic cases (*p*-value = 0.021), and in ER-negative versus ER-positive cases (*p*-value = 0.0005, Table 2). The excess of PTV carriers in familial cases with respect to sporadic cases has been shown for other genes established as moderate-risk factors for breast cancer, for example *CHEK2*. Specifically, the C*HEK2*:c.1100delC PTV, which accounts for the majority of *CHEK2* PTVs, has been shown to have a 2.79-fold higher frequency or to confer a 1.77-fold higher risk in familial versus sporadic breast cancer cases [19,20]. *FANCM* has been reported as specifically associated with ER-negative breast cancer risk [13]. Hence, the excess of *FANCM* PTVs that we observed in familial cases and in ER-negative cases is reinforcing the knowledge that *FANCM* is a moderate-risk gene for breast cancer. Importantly, the fact that frequency differences increased after the exclusion of p.Gln1701* and p.Gly1906Alafs*12 carriers corroborates the hypothesis that these two PTVs have a lower impact on breast cancer risk.

### 3.2. Spectrum of Common and Rare FANCM PTVs

**Figure 2:**
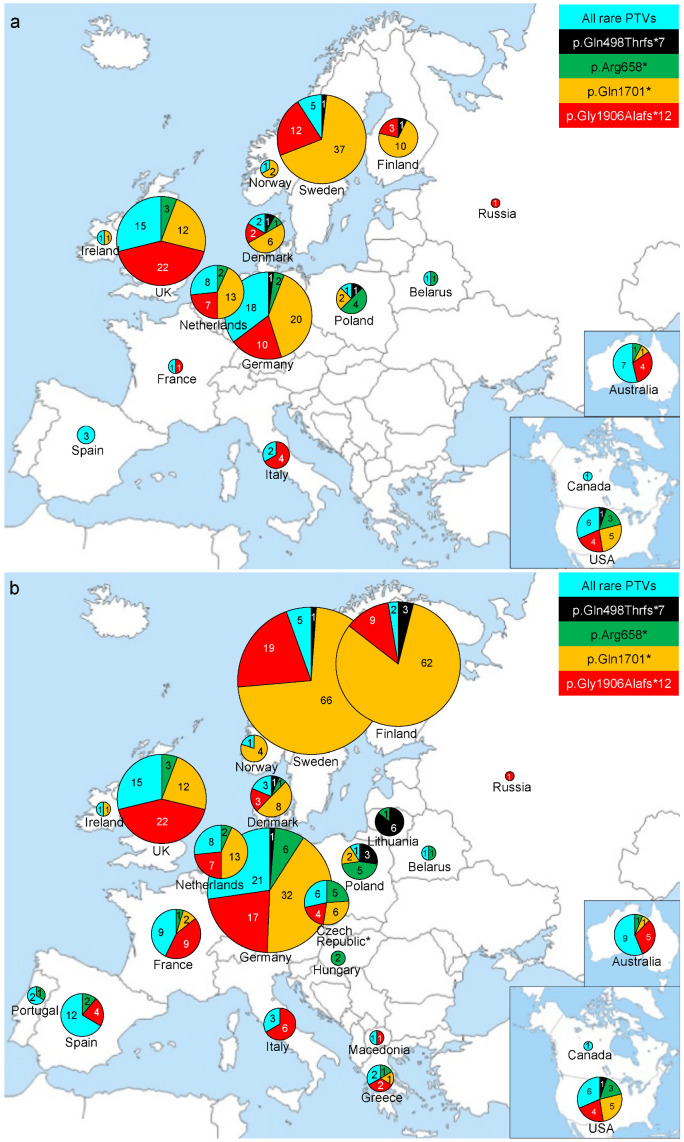
Geographic spectrum of common and rare *FANCM* PTVs presented by absolute number of PTV carriers identified per country. The pie charts sizes represent the total number of carriers per country. (**a**) Spectrum of 66 different *FANCM* PTVs found in 274 carriers by sequencing of 44,803 breast cancer cases in the present study. (**b**) Spectrum of 91 different *FANCM* PTVs found in a total of 487 carriers obtained by combining data from the present study with those we published previously (Figlioli et al., 114 carriers from several countries [18]) and with all the other available studies based on the *FANCM* sequencing in breast cancer cases (Cavaillé et al., 4 carriers from France [24]; Del Valle et al., 2 carriers from Spain [25]; Helgadottir et al., 4 carriers from Sweden [26]; Jarhelle et al., 2 carriers from Norway [27]; Neidhardt et al., 21 carriers from Germany [28]; Nurmi et al., 58 carriers from Finland [29]; Schubert et al., 5 carriers from Germany [30]; Southey et al., 3 carriers from Australia [31]). * One individual is a bi-allelic carrier of *FANCM* PTVs.

Among the 66 different variants, four, namely p.Gln498Thrfs*7 (*FANCM*:c.1491dupA), p.Arg658*, p.Gln1701* and p.Gly1906Alafs*12, were relatively common, each being identified in at least six carriers. The remaining 62 variants were unique or were found in a maximum of three carriers and were classified as “rare *FANCM* PTVs” (Figure 1, Supplementary Table S2). Of the 274 carriers, 202 (73.7%) carried one of the four common *FANCM* PTVs. Of these 202 carriers, 6 (3.0%), carried p.Gln498Thrfs*7, 17 (8.4%) carried p.Arg658*, 109 (54.0%) carried p.Gln1701* and 70 (34.6%) carried p.Gly1906Alafs*12. The remaining 72 carriers (26.3% of the total) carried one of the 62 rare PTVs (Figure 2a). Of the 62 rare PTVs, 54 were unique, six were found in two breast cancer cases, and two in three breast cancer cases (Supplementary Table S2). These results were consistent with those of a previous study in which we described the spectrum of 27 different *FANCM* PTVs identified in 114 European female breast cancer cases [18]. In fact, we observed that p.Gln1701* was the most common PTV in Northern Europe, with highest frequencies in Finland and Sweden and decreasing frequencies along the North-South axis (Figure 2a). Similarly, p.Gly1906Alafs*12 was validated to be the most common PTV in Southern Europe with decreasing frequencies in Central and Northern Europe. We also confirmed that p.Arg658* was the third most common PTV which was common in Central Europe with higher frequencies in Eastern Europe. Moreover, the geographical origin of the six p.Gln498Thrfs*7 carriers was compatible with our previous findings indicating that this PTV is probably a founder variant from Lithuania [18] (Figure 2a). Furthermore, with respect to the distribution of rare PTVs, it appears that carrier frequencies in Germany and Sweden are higher than those we previously reported [18] (Figure 2a). Finally, we observed heterogeneous spectra in Australia and USA consistent with the fact that those carriers are of European ancestry.

### 3.3. Comprehensive Spectrum of FANCM PTVs

We combined the here presented data with those we published previously [18], and with all the other available studies based on *FANCM* sequencing of European breast cancer cases [24,25,26,27,28,29,30,31]. Figure 2b shows the distribution spectrum of a total of 91 different *FANCM* PTVs found in 487 breast cancer cases from 23 countries. This map shows the different frequency distributions and the specific prevalence of p.Gln498Thrfs*7, p.Arg658*, p.Gln1701* and p.Gly1906Alafs*12 PTVs. It could be also observed that the spectra of *FANCM* PTVs seem to be much more heterogeneous in Southwestern Europe (i.e., Portugal, Spain and France) with respect to Northeastern Europe (i.e., Sweden, Finland and Norway (Figure 2b)). To investigate this observation better, we grouped the tested countries in those from Southwestern or Central Europe (Portugal, Spain, Italy, Greece, Macedonia, Hungary, Czech Republic, Germany, France, the Netherlands, UK and Ireland) and those from Northeastern Europe (Finland, Sweden, Norway, Denmark, Poland, Lithuania, Belarus and Russia). If we consider the carriers of rare PTVs, there were 80 (33.1% of 242 total carriers) in Southwestern and Central Europe versus only 13 (6.2% of 209 total carriers) in Northeastern Europe (*p*-value < 0.0001). Considering specifically the single different PTVs, we observed that there were 62 (25.6% of 242 carriers) in Southwestern and Central Europe compared with 16 (7.6% of 209 carriers) in Northeastern Europe (*p*-value < 0.0001). Only for some of the 87 rare different PTVs, it was possible to speculate on the geographic origin. In particular, we considered the eight PTVs that were found in at least three carriers (Table 3). Among these, p.Arg185Glufs*13 and p.Gln498* might be prevalent in Germany and the Netherlands, while p.Glu774* and p.Lys863Ilefs*12 might be specific to the Iberian Peninsula, and to Spain and France, respectively. Finally, p.Tyr1398* could be from the UK. However, since we could not exclude that some of these carriers were originally members of the same family that were ascertained as different probands, additional data are required to confirm these hypotheses.

## 4. Conclusions

In this study, we report *FANCM* PTV carrier frequencies among 44,803 breast cancer cases from 19 countries. In addition, our data in combination with data from previous studies allowed us to describe the spectra of 91 *FANCM* PTVs in breast cancer cases from Europe, USA, Canada and Australia. These data could be used to inform first pass genotyping screening, and for more efficient genetic testing strategies in breast cancer cases from specific populations.

## Figures and Tables

**Table 1:** Frequencies of all protein truncating variants (PTVs) carriers and those excluding p.Gln1701* and p.Gly1906Alafs*12 found in 44,803 breast cancer cases from 19 countries.

Country	Breast Cancer Cases	Carriers of All PTV (Freq%)	Carriers of all PTV Excluding p.Gln1701* and p.Gly1906Alafs*12 (Freq%)
UK	10,683	52 (0.9)	18 (0.17)
Germany	8659	51 (0.59)	21 (0.24)
Sweden	4607	55 (1.19)	6 (0.13)
Netherlands	3705	30 (0.81)	10 (0.27)
USA	2800	19 (0.68)	10 (0.36)
Denmark	2800	12 (0.43)	4 (0.14)
Australia	2460	13 (0.53)	8 (0.32)
Poland	2103	8 (0.38)	6 (0.28)
Spain	1126	3 (0.27)	3 (0.27)
Cyprus	974	0 (0)	0 (0)
France	938	2 (0.21)	1 (0.11)
Italy	933	6 (0.64)	2 (0.21)
Norway	565	3 (0.53)	1 (0.18)
Finland	560	14 (2.50)	1 (0.178)
Canada	491	1 (0.20)	1 (0.20)
Greece	472	0 (0)	0 (0)
Ireland	369	2 (0.54)	1 (0.27)
Belarus	319	2 (0.63)	2 (0.63)
Russia	239	1 (0.42)	0 (0)
Total	44,803	274 (0.61)	95 (0.21)

Freq, frequency.

**Table 2:** Frequencies of all protein truncating variants (PTVs) carriers and those excluding p.Gln1701* and p.Gly1906Alafs*12 found in 44,803 breast cancer cases grouped by their breast cancer family history and the ER status.

Breast Cancer Cases		Carriers of All PTV (Freq%)	*p*-Value	Carriers of All PTV Excluding p.Gln1701* and p.Gly1906Alafs*12 (Freq%)	*p*-Value
Group	Number				
All	44,803	274 (0.61)	-	95 (0.21)	-
Sporadic	26,539	150 (0.56)	0.032	49 (0.18)	0.021
Familial	10,680	81 (0.76)		33 (0.31)	
ER-positive	25,679	137 (0.53)	0.048	44 (0.17)	0.0005
ER-negative	6572	49 (0.74)		26 (0.39)	

**Table 3:** List of *FANCM* rare protein truncating variants (PTVs) that, through combining data from the present study with those from previously published studies, were identified in at least three individuals. For each PTV, the total number of detected carriers is reported along with the study/database and the country of origin.

PTV	Number of Carriers	Study, Geographic Origin
c.551dup; p.Arg185Glufs*13	3	This study, Germany (1) Netherlands (1); Neidhardt et al. [28], Germany (1)
c.1196C>G; p.Ser399*	3	This study, UK (2), Figlioli et al. [18], Spain (1)
c.1492C>T; p.Gln498*	3	This study, Germany (1) Netherlands (2)
c.2260C>T; p.Arg754*	3	Figlioli et al. [18], Czech Republic (2) France (1)
c.2320G>T; p.Glu774*	4	This study, Spain (1); Figlioli et al. [18], Spain (2) Portugal (1)
c.2586_2589del; p.Lys863Ilefs*12	3	This study, Spain (1); Figlioli et al. [18], Spain (1); Cavaillé et al. [24], France (1)
c.3088C>T; p.Arg1030*	3	This study, Spain (1); Figlioli et al. [18], Czech Republic (1); Neidhardt et al. [28], Germany (1)
c.4194T>G; p.Tyr1398*	3	This study, UK (3)

## Data Availability

The data presented in this study are available on request to the BCAC Data Access Co-ordinating Committee (bcac@medschl.cam.ac.uk). The data are not publicly available due to privacy or ethical restrictions. Summary individual-level data are available in Supplementary Table S2.

## Supplementary Materials

The following supporting information can be downloaded at: https://www.mdpi.com/article/10.3390/cancers15133313/s1, Supplementary Table S1: Description of the 39 studies included in the present analysis. Supplementary Table S2: List of the 274 breast cancer cases carrying one of the 66 *FANCM* protein truncating variants (PTVs).

## References

[1] Lilyquist J., Ruddy K.J., Vachon C.M., Couch F.J. (2018). Common Genetic Variation and Breast Cancer Risk-Past, Present, and Future. Cancer Epidemiol. Biomark. Prev..

[2] Kuchenbaecker K.B., Hopper J.L., Barnes D.R., Phillips K.A., Mooij T.M., Roos-Blom M.J., Jervis S., van Leeuwen F.E., Milne R.L., Andrieu N. (2017). Risks of Breast, Ovarian, and Contralateral Breast Cancer for BRCA1 and BRCA2 Mutation Carriers. JAMA.

[3] Yang X., Leslie G., Doroszuk A., Schneider S., Allen J., Decker B., Dunning A.M., Redman J., Scarth J., Plaskocinska I. (2020). Cancer Risks Associated With Germline PALB2 Pathogenic Variants: An International Study of 524 Families. J. Clin. Oncol..

[4] Dorling L., Carvalho S., Allen J., Gonzalez-Neira A., Luccarini C., Wahlstrom C., Pooley K.A., Parsons M.T., Fortuno C., Breast Cancer Association Consortium (2021). Breast Cancer Risk Genes—Association Analysis in More than 113,000 Women. N. Engl. J. Med..

[5] Hu C., Hart S.N., Gnanaolivu R., Huang H., Lee K.Y., Na J., Gao C., Lilyquist J., Yadav S., Boddicker N.J. (2021). A Population-Based Study of Genes Previously Implicated in Breast Cancer. N. Engl. J. Med..

[6] Laitman Y., Friebel T.M., Yannoukakos D., Fostira F., Konstantopoulou I., Figlioli G., Bonanni B., Manoukian S., Zuradelli M., Tondini C. (2019). The spectrum of BRCA1 and BRCA2 pathogenic sequence variants in Middle Eastern, North African, and South European countries. Hum. Mutat..

[7] Rebbeck T.R., Friebel T.M., Friedman E., Hamann U., Huo D., Kwong A., Olah E., Olopade O.I., Solano A.R., Teo S.H. (2018). Mutational spectrum in a worldwide study of 29,700 families with BRCA1 or BRCA2 mutations. Hum. Mutat..

[8] Janssen B., Bellis S., Koller T., Tischkowitz M., Liau S.S. (2020). A systematic review of predicted pathogenic PALB2 variants: An analysis of mutational overlap between epithelial cancers. J. Hum. Genet..

[9] Suszynska M., Kozlowski P. (2020). Summary of BARD1 Mutations and Precise Estimation of Breast and Ovarian Cancer Risks Associated with the Mutations. Genes.

[10] Boni J., Idani A., Roca C., Feliubadalo L., Tomiak E., Weber E., Foulkes W.D., Orthwein A., El Haffaf Z., Lazaro C. (2022). A decade of RAD51C and RAD51D germline variants in cancer. Hum. Mutat..

[11] Pavlovica K., Irmejs A., Noukas M., Palover M., Kals M., Tonisson N., Metspalu A., Gronwald J., Lubinski J., Murmane D. (2022). Spectrum and frequency of CHEK2 variants in breast cancer affected and general population in the Baltic states region, initial results and literature review. Eur. J. Med. Genet..

[12] Foulkes W.D. (2021). The ten genes for breast (and ovarian) cancer susceptibility. Nat. Rev. Clin. Oncol..

[13] Peterlongo P., Figlioli G., Deans A.J., Couch F.J. (2021). Protein truncating variants in FANCM and risk for ER-negative/triple negative breast cancer. NPJ Breast Cancer.

[14] Figlioli G., Bogliolo M., Catucci I., Caleca L., Lasheras S.V., Pujol R., Kiiski J.I., Muranen T.A., Barnes D.R., Dennis J. (2019). The FANCM:p.Arg658* truncating variant is associated with risk of triple-negative breast cancer. NPJ Breast Cancer.

[15] Peterlongo P., Catucci I., Colombo M., Caleca L., Mucaki E., Bogliolo M., Marin M., Damiola F., Bernard L., Pensotti V. (2015). FANCM c.5791C>T nonsense mutation (rs144567652) induces exon skipping, affects DNA repair activity and is a familial breast cancer risk factor. Hum. Mol. Genet..

[16] Kiiski J.I., Pelttari L.M., Khan S., Freysteinsdottir E.S., Reynisdottir I., Hart S.N., Shimelis H., Vilske S., Kallioniemi A., Schleutker J. (2014). Exome sequencing identifies FANCM as a susceptibility gene for triple-negative breast cancer. Proc. Natl. Acad. Sci. USA.

[17] Kiiski J.I., Tervasmaki A., Pelttari L.M., Khan S., Mantere T., Pylkas K., Mannermaa A., Tengstrom M., Kvist A., Borg A. (2017). FANCM mutation c.5791C>T is a risk factor for triple-negative breast cancer in the Finnish population. Breast Cancer Res. Treat..

[18] Figlioli G., Kvist A., Tham E., Soukupova J., Kleiblova P., Muranen T.A., Andrieu N., Azzollini J., Balmana J., Barroso A. (2020). The Spectrum of FANCM Protein Truncating Variants in European Breast Cancer Cases. Cancers.

[19] CHEK2 Breast Cancer Case-Control Consortium (2004). CHEK2*1100delC and susceptibility to breast cancer: A collaborative analysis involving 10,860 breast cancer cases and 9,065 controls from 10 studies. Am. J. Hum. Genet..

[20] Weischer M., Bojesen S.E., Ellervik C., Tybjaerg-Hansen A., Nordestgaard B.G. (2008). CHEK2*1100delC genotyping for clinical assessment of breast cancer risk: Meta-analyses of 26,000 patient cases and 27,000 controls. J. Clin. Oncol..

[21] Deans A.J., West S.C. (2009). FANCM connects the genome instability disorders Bloom’s Syndrome and Fanconi Anemia. Mol. Cell.

[22] UniProt C. (2021). UniProt: The universal protein knowledgebase in 2021. Nucleic Acids Res..

[23] Walden H., Deans A.J. (2014). The Fanconi anemia DNA repair pathway: Structural and functional insights into a complex disorder. Annu. Rev. Biophys..

[24] Cavaille M., Uhrhammer N., Privat M., Ponelle-Chachuat F., Gay-Bellile M., Lepage M., Molnar I., Viala S., Bidet Y., Bignon Y.J. (2021). Analysis of 11 candidate genes in 849 adult patients with suspected hereditary cancer predisposition. Genes Chromosomes Cancer.

[25] Del Valle J., Rofes P., Moreno-Cabrera J.M., Lopez-Doriga A., Belhadj S., Vargas-Parra G., Teule A., Cuesta R., Munoz X., Campos O. (2020). Exploring the Role of Mutations in Fanconi Anemia Genes in Hereditary Cancer Patients. Cancers.

[26] Helgadottir H.T., Thutkawkorapin J., Lagerstedt-Robinson K., Lindblom A. (2021). Sequencing for germline mutations in Swedish breast cancer families reveals novel breast cancer risk genes. Sci. Rep..

[27] Jarhelle E., Riise Stensland H.M.F., Hansen G.A.M., Skarsfjord S., Jonsrud C., Ingebrigtsen M., Stromsvik N., Van Ghelue M. (2019). Identifying sequence variants contributing to hereditary breast and ovarian cancer in BRCA1 and BRCA2 negative breast and ovarian cancer patients. Sci. Rep..

[28] Neidhardt G., Hauke J., Ramser J., Gross E., Gehrig A., Muller C.R., Kahlert A.K., Hackmann K., Honisch E., Niederacher D. (2017). Association Between Loss-of-Function Mutations Within the FANCM Gene and Early-Onset Familial Breast Cancer. JAMA Oncol..

[29] Nurmi A.K., Suvanto M., Dennis J., Aittomaki K., Blomqvist C., Nevanlinna H. (2022). Pathogenic Variant Spectrum in Breast Cancer Risk Genes in Finnish Patients. Cancers.

[30] Schubert S., van Luttikhuizen J.L., Auber B., Schmidt G., Hofmann W., Penkert J., Davenport C.F., Hille-Betz U., Wendeburg L., Bublitz J. (2019). The identification of pathogenic variants in BRCA1/2 negative, high risk, hereditary breast and/or ovarian cancer patients: High frequency of FANCM pathogenic variants. Int. J. Cancer.

[31] Southey M.C., Dowty J.G., Riaz M., Steen J.A., Renault A.L., Tucker K., Kirk J., James P., Winship I., Pachter N. (2021). Population-based estimates of breast cancer risk for carriers of pathogenic variants identified by gene-panel testing. NPJ Breast Cancer.

