# Supplementary material for: Spectrum and Frequency of Germline FANCM Protein-Truncating Variants in 44,803 European Female Breast Cancer Cases
Source: Cancers (Basel). 2023 Jun 23;15(13):3313. doi: 10.3390/cancers15133313 (PMC10340689; doi:10.3390/cancers15133313)
Supplement: Supplementary file 1 [file cancers-15-03313-s001.zip › cancers-2378077-supplementary/Suppl_table_S1.pdf]

Table S1. Description of the 39 studies included in the present analysis.

Study	Abbreviation	Country	Study design	Number of breast cancer tested	No. of FANCM PTV carriers
Amsterdam Breast Cancer Study	ABCS	Netherlands	Hospital-based consecutive cases; population-based controls	1,007	9
Amsterdam Breast Cancer Study - Familial	ABCS-F	Netherlands	Clinical Genetic Center-based cases	208	2
Bavarian Breast Cancer Cases and Controls	BBCC	Germany	Hospital-based cases; population based controls	243	2
Breast Cancer in Galway Genetic Study	BIGGS	Ireland	Hospital-based cases; population based controls	369	2
Breast Oncology Galicia Network	BREOGAN	Spain	Population-based case-control	595	3
Breast Cancer Study of the University of Heidelberg	BSUCH	Germany	Hospital-based cases;healthy blood donator controls	241	1
Crete Cancer Genetics Program	CCGP	Greece	Hospital-based case-control study	472	0
CECILE Breast Cancer Study	CECILE	France	Population-based case-control study	938	2
Copenhagen General Population Study	CGPS	Denmark	Population-based case-control study	2,800	12
Spanish National Cancer Centre Breast Cancer Study	CNIO-BCS	Spain	Case-control study	531	0
Family History Risk Study	FHRISK	UK	Clinic-based cohort study with a nested case-control study	79	1
German Consortium for Hereditary Breast & Ovarian Cancer	GC-HBOC	Germany	Clinic-based case study and prospective cohort study	2,565	20
Gene Environment Interaction and Breast Cancer in Germany	GENICA	Germany	Population-based case-control study	848	9
Generation Scotland	GENSCOT	Scotland	Prospective family-based cohort study; nested case-control	427	0
Genetic Epidemiology Study of Breast Cancer by Age 50	GESBC	Germany	Population-based study of women <50 years	547	4
Hannover Breast Cancer Study	HABCS	Germany	Hospital-based case-control study	967	1
Hereditair Borst-en eierstokkanker Onderzoek Nederland	HEBON	Netherlands	Clinical genetic center-based recruitment of familial breast or ovarian cancer patients (cases)	1,453	12
Hannover- Minsk Breast Cancer Study	HMBCS	Belarus	Hospital-based cases; population based controls	319	2
Hannover-Ufa Breast Cancer Study	HUBCS	Russia	Hospital-based cases; population based controls	239	1
Karolinska Breast Cancer Study	KARBAC	Sweden	Population and hospital-based cases; geographically matched controls	366	4
Karolinska Mammography Project for Risk Prediction of Breast Cancer - Cohort Study	KARMA	Sweden	Cohort study	3,087	37
Kuopio Breast Cancer Project	KBCP	Finalnd	Population-based prospective clinical cohort	560	14

Kathleen Cuningham Foundation Consortium for research into Familial Breast Cancer/Australian Ovarian Cancer Study	KCONFAB/AOCS	Australia and New Zealand	Clinic-based recruitment of familial breast cancer patients (cases); population-based case-control study of ovarian cancer (controls only)	1,418	8
Mammary Carcinoma Risk Factor Investigation	MARIE	Germany	Population-based case-control study	2,295	10
Cyprus Breast Cancer Case Control Study	MASTOS	Cyprus	Population-based case-control study	974	0
Milan Breast Cancer Study Group	MBCSG	Italy	Clinic-based recruitment of familial/early onset breast cancer patients (cases); population-based controls	933	6
Melbourne Collaborative Cohort Study	MCCS	Australia	Prospective cohort study: nested case-control study	1,042	5
Norwegian Breast Cancer Study	NBCS	Norway	Hospital-based case-control study	565	3
Ontario Familial Breast Cancer Registry	OFBCR	Canada	Population-based familial case-control study	491	1
NCI Polish Breast Cancer Study	PBCS	Poland	Population-based case-control study	1,757	7
Karolinska Mammography Project for Risk Prediction of Breast Cancer - Case-Control Study	PKARMA	Sweden	Population-based study	23	0
The Prostate,Lung,Colorectal and Ovarian (PLCO) Cancer Screening Trial	PLCO	USA	Prospective cohort study: nested case-control	2,041	10
Predicting the Risk Of Cancer At Screening Study	PROCAS	UK	Population based study	495	4
Rotterdam Breast Cancer Study	RBCS	Netherlands	Clinical Genetic Center-based case-control study, Rotterdam area	1,037	7
Singapore and Sweden Breast Cancer Study	SASBAC	Sweden	Population-based case-control study	1,131	14
Study of Epidemiology and Risk factors in Cancer Heredity	SEARCH	UK	Population-based case-control study	9,682	47
Städtisches Klinikum Karlsruhe Deutsches Krebsforschungs zentrum Study	SKKDKFZS	Germany	Hospital-based breast cancer cohort	953	4
IHCC-Szczecin Breast Cancer Study	SZBCS	Poland	Hospital-based case-control study	346	1
Utah Breast Cancer Study	UBCS	USA	Population-based and hospital-based case-control study, plus additional familial cases	759	9
All studies				44,803	274
